# Supplementary material for: Early Oral Administration of Ginseng Stem-Leaf Saponins Enhances the Peyer’s Patch-Dependent Maternal IgA Antibody Response to a PEDV Inactivated Vaccine in Mice, with Gut Microbiota Involvement
Source: Vaccines (Basel). 2023 Apr 12;11(4):830. doi: 10.3390/vaccines11040830 (PMC10143706; doi:10.3390/vaccines11040830)
Supplement: Supplementary file 1 [file vaccines-11-00830-s001.zip › vaccines-2299950-supplementary.pdf]

Supplementary Material

Table S1. Up-regulated genes related to tight junction, intestinal immune network for IgA production RNA seq profiling in the mammary gland of the mice orally administered with GSLS before vaccination.

Gene name	Gene description	GSLS_1	GSLS_2	GSLS_3	PEDV_1	PEDV_2	PEDV_3
Tight junction							
Cd1d1	CD1d1 antigen [Source:MGI Symbol;Acc:MGI:107674]	10.57	7.42	11.24	3.84	3.34	1.96
Myh11	myosin, heavy polypeptide 11, smooth muscle [Source:MGI Symbol;Acc:MGI:102643]	31.88	33.86	32.13	17.14	15.75	20.97
Map3k5	mitogen-activated protein kinase kinase kinase 5 [Source:MGI Symbol;Acc:MGI:1346876]	3.79	3.50	3.94	1.84	2.49	2.42
Hcls1	hematopoietic cell specific Lyn substrate 1 [Source:MGI Symbol;Acc:MGI:104568]	1.89	2.32	1.53	0.32	0.46	0.82
Intestinal immune network for IgA production							
Tnfrsf13b	tumor necrosis factor receptor superfamily, member 13b [Source:MGI Symbol;Acc:MGI:1889411]	5.26	3.65	2.84	2.00	1.04	1.85
Ccr10	chemokine (C-C motif) receptor 10 [Source:MGI Symbol;Acc:MGI:1096320]	0.87	1.06	0.70	0.38	0.15	0.09
Tnfsf13	tumor necrosis factor (ligand) superfamily, member 13 [Source:MGI Symbol;Acc:MGI:1916833]	6.64	5.81	5.35	2.13	2.04	1.43
Tgfb1	transforming growth factor, beta 1 [Source:MGI Symbol;Acc:MGI:98725]	5.51	7.35	5.67	3.19	4.13	3.21
